# Rapid reduction in migration distance in relation to climate in a long-distance migratory bird

**DOI:** 10.1093/cz/zoab053

**Published:** 2021-07-21

**Authors:** Anders Pape Møller, Tim van Nus, Keith A Hobson

**Affiliations:** Ecologie Systématique Evolution, Université Paris-Sud, CNRS, AgroParisTech, Université Paris-Saclay, Orsay Cedex F-91405, France; Ministry of Education, Key Laboratory for Biodiversity Science and Ecological Engineering, College of Life Sciences, Beijing Normal University, Beijing 100875, China; Chasséstraat 34-2, Amsterdam NL-1057JG, The Netherlands; Department—Biology and Environment and Climate Change Canada, University of Western Ontario, Room 2025 BGS Building, 1151 Richmond Street, London, ON, Canada N6A 5B7

Migratory birds likely evolved from residents as a consequence of intraspecific competition for limiting resources followed by dispersal of such individuals to novel sites that had previously not been occupied by migrant conspecifics (Alerstam and Högstedt 1982). If recent climate change has increased the benefits of short-distance migration and residence, with such individuals not paying the fitness costs of longer migration, we should expect an increase in the abundance of these shorter-distance migrants or residents. Alternatively, seasonal fluctuations in food may result in intraspecific competition or altered patterns of avoidance of predators and parasites that may affect the relative role of migration and residency for the distribution of barn swallows (*Hirundo rustica*; Cheng et al. 2019). Barn swallows are long-distance migratory birds with a global breeding distribution with resident populations in Egypt and possibly in south China (eBird back to 1993 and http://datazone.birdlife.org/species/factsheet/barn-swallow-hirundo-rustica) and are increasingly wintering further northward from the equator due to climate change. Studies of stable hydrogen isotopes in feathers (*δ*^2^H_f_) grown in natal areas and at the wintering grounds can be used to infer molt origins and have shown that barn swallows are philopatric to their wintering grounds, and during winter to their once-chosen communal winter roosts (Szép et al. 2009). During the last 5 years, hundreds of individual barn swallows have increasingly abandoned long-distance migration and have instead wintered closer to their breeding grounds in Southern Europe (BirdLife International 2021). There is also information on change in timing of molt in barn swallows in South Africa, and this has had consequences for the northward shift in molt and migration by barn swallows wintering in that area (Ambrosini et al. 2011). Barn swallows have recently started to winter in Porto, Lisbon, and Algarve, Portugal, during December–February (van Nus and Neto 2017). The change in migratory behavior by large numbers of individual barn swallows in southern Europe constitutes a reduction in migratory distance and the timing of the annual molt. Here, we first used measurements of *δ*^2^H_f_ from Portuguese-wintering barn swallows during the annual molt to assign molt origins to a mixed sample of juvenile and adult birds (Hobson et al. 2012). We used characteristics of tail feather length and the size of white spots on these feathers to estimate the proportion of adults and juvenile birds in our sample (Møller et al. 1995; Kose and Møller 1999; Supplementary Material). Growth bars were used to estimate feather growth rates (Grubb 2006; Supplementary Material). However, we were forced to use only primary feathers for the isotopic analysis and subsequent depiction of molt origins using the isotope approach (Supplementary Material). We assumed that the proportion of adults versus juvenile birds determined from tail feathers would be approximately the same as the distribution of these 2 age classes in the primary feathers for the isotope sample. The *δ*^2^H_f_ values allowed us to estimate natal origins in Europe of juvenile barn swallows, and if adults had grown feathers the previous year in the traditional west African or European winter quarters in Europe. If adults had grown feathers in Europe versus Africa, this would indicate an established wintering population in Europe that had abandoned long-distance migration to Africa. Second, we tested whether molt phenology of barn swallows that have recently started to winter in Portugal differed from breeding birds that originated from the Iberian breeding grounds while wintering in Western Africa.

We found a total of 561 feathers of which 332 were primaries, 214 tail feathers, and 15 secondaries. Our analyses of tail feathers revealed that the approximate proportion of adults in the population was 66.5%. The length of primaries and secondaries was normally distributed, while the length of the outermost tail feathers was bimodally distributed. The category of long tail feathers (typical of adults) was on average 92.46 mm (*SE* = 1.15 mm), *N = *50, while the category of short-tail feathers (typical of juveniles) was 61.85 mm (*SE* = 0.69 mm), *N = *141. Molt during 2015–2016 in Portugal took place during 4 months between November and February. Using growth patterns, feathers in swallows in Portugal grew 2.82 mm/day (*SE* = 0.011 mm), *N = *214 feathers. There was a significant difference in duration of feather molt between years ([LR = 4.76, *df* *=* 1, *P = *0.029], estimate [*SE*] = −4.052 days [0.029]). Measurements of naturally occurring deuterium (^2^H) in the plumage of barn swallows wintering in Portugal overwhelmingly confirmed European natal origins of juvenile birds and showed that the majority of adults had also grown their feathers the previous year in Europe rather than at their traditional sub-Saharan African winter quarters (Figure 1 and Supplementary Table S1). We showed that for all feathers analyzed, δ^2^H_f_ was on average −57.0‰, SE = 1.35‰, range −27.4‰ to −92.8‰, *N = *69. Duration of feather molt that was independent of age decreased with increasing *δ*^2^H_f_, with more southerly grown feathers in Europe ([LR = 8.06, *df* *=* 1, *P *<* *0.0001], mean estimate [SE] = −23.67‰ [8.10‰]). Feather shaft length increased with *δ*^2^H_f_ (LH = 4.57, df *=* 1, *P = *0.033, estimate [SE] = 0.65 mm [0.30]). The date when individuals molted feathers advanced with *δ*^2^H_f_ (LR = 13.47, *df* *=* 1, *P* < 0.0001, estimate [*SE*] = 1.22 days [0.32]). Thus, duration of molt increased with *δ*^2^H_f_ while early phenology of molt advanced with *δ*H_f_. Tail length decreased with *δ*^2^H_f_ in a model that controlled for feather tract (whether feathers were from wing or tail; LR = 34.32, *df* *=* 1, *P* < 0.0001, estimate [*SE*] = −0.000925 [0.0002657 mm]). In contrast, tail feather width of the same feathers was more weakly related to tail feather length and *δ*^2^H_f_ (LR = 3.76, *df* *=* 1, *P = *0.053, estimate [*SE*] = −0.028 mm [0.014 mm]). This implies that feather dimensions decreased with *δ*^2^H_f_, or with more southerly grown feathers in Europe. We examined trends in temperatures at Aveiro over 11 years. Minimum temperatures increased from 4.3 °C to 9.3 °C during December–January 2009–2019 and maximum temperatures from 24.4 °C to 28.4 °C at the local weather station at Aveiro, Portugal. The temperature in December–January during 2009–2019 increased from 5.8 °C in 2008 (95% confidence intervals [CIs]: 4.8–6.8 °C) to 7.9 °C in 2019 (95% CI: 6.9–8.9 °C). This increase was statistically significant (GLM with normally distributed data and an identity link function; likelihood ratio statistic LR = 4.47, df *=* 1, *P = *0.035, estimate [*SE*] = 0.208 [0.089]).

**Figure 1.**
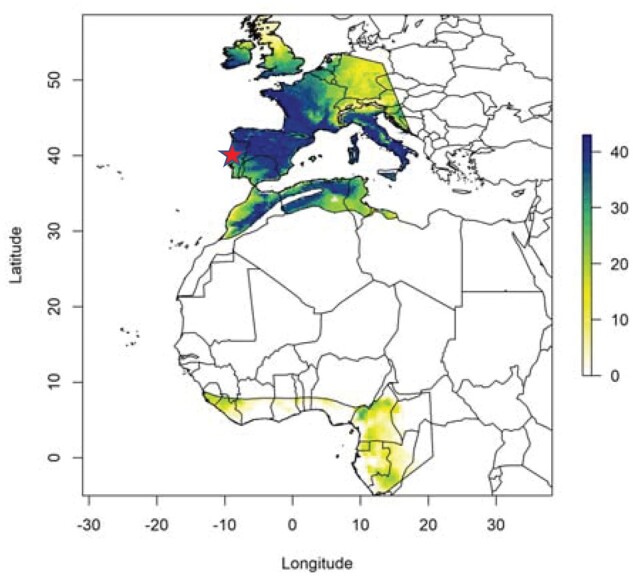
Depiction of molt origins of barn swallows based on *δ*^2^H values in distal primary vane measurements for 2016 and 2017 (no year effects in assignment observed). The number of individuals associated with each pixel of the map having a probability of occurrence equal to or greater than 67%. Potential origins were bounded by western Europe on the breeding grounds and assumed western sub-Saharan wintering grounds of west Africa. Colors ranging from white to dark blue reflect higher probability of origin. The roost in Aveiro is shown with a red star.

Assignment of barn swallows to their molting grounds based on primary feathers only indicated support for winter molt (the previous and subsequent year) largely in Europe with some birds possibly molting as far north as France in the preceding year and a smaller group having molted previously in Africa (Figure 1). Within Europe-grown feathers, there was a significant difference in *δ*^2^H_f_ with date, showing that birds that molted later also wintered more northward in the previous year.

Numerous species of migratory birds have been shown to advance the timing of migration phenology in recent decades. Traditionally, barn swallows from Spain and Portugal molt during 5 months in September–January in western Africa (Møller 1994) before migrating back to the breeding sites in the Iberian Peninsula in February–April (Møller 1994). The timing and the long duration of the single annual molt suggests that barn swallows cannot migrate to Western Africa in January–February before returning to the breeding grounds in the Iberian Peninsula (Møller 1994). Based on our isotope data and detailed analyses of feather growth patterns, we conclude that barn swallows have recently changed their migration from breeding areas in the Iberian Peninsula to now also winter in the Iberian Peninsula. Our examination of the mean winter temperature in Portugal showed increases over the last decade confirming that barn swallows wintering there is consistent with a response to climate change in general and warmer winters in particular.

## Ethics

The study did not require ethics approval since only molted feathers were used.

## Data Accessibility

All data are available in the manuscript.

## Authors’ Contributions

A.P.M. and K.A.H. designed the study and wrote the manuscript. T.v.N. collected the samples. K.A.H. analyzed the samples. A.P.M. analyzed the data.

## Supplementary Material

zoab053_Supplementary_Material
