# Supplementary material for: Rapid reduction in migration distance in relation to climate in a long-distance migratory bird
Source: Curr Zool. 2021 Jul 21;68(2):233–5. doi: 10.1093/cz/zoab053 (PMC8962680; doi:10.1093/cz/zoab053)
Supplement: zoab053_Supplementary_Material [file zoab053_supplementary_material.docx]

**Supplementary Material**

**Letter to the Editor**

**Rapid reduction in migration distance in relation to climate in a long-distance migratory bird**

Anders Pape Møller ^1,2^, Tim van Nus^3^ and Keith A. Hobson^4^

**Background**

van Nus and Neto (2017) identified a communal roost with up to 150 moulting barn swallows inside the town of Aveiro (40º38' N, 8º39' W), Portugal during the winters (November-February) 2015-2016 and 2016-2017. The birds were roosting on electricity cables and window frames inside the town (photos in van Nus and Neto 2017). Barn swallows from Aveiro were recorded foraging up to 8 km from the roost, suggesting that these birds were moulting winter residents that were foraging in the neighbourhood of Aveiro rather than them being migrants that had arrived from elsewhere. The birds abandoned the roost at the end of January coinciding with the end of the moult and the start of the breeding season for barn swallows in Portugal and Western and Southern Spain (Møller 1994), but much earlier than in other European breeding populations. This implies that barn swallows recorded roosting at and abandoning the roosting site at Aveiro by the end of January must have been breeding in Portugal and south western Spain since these areas are the only ones with populations breeding in the southwestern part of the Iberian Peninsula (Møller 1994). Multiple feathers could originate from a single individual although that is unlikely given the many hundreds of individuals largely philopatric to this site (see Szép et al. 2009) and so we used our feather sample to indicate the proportion of adults and juveniles in the sampled population for stable isotope analyses.

Barn swallows of Central and Northern Europe initiate moult in Sub-Saharan Africa during a prolonged period of 5-6 months (Møller et al. 1995). The timing of moult by populations in South Africa has resulted in later spring migration in recent years and altered phenology of migration. Moulted feathers in Aveiro, Portugal were found on the ground below the roost during regular visits at intervals of 1-3 days during the period from 20 December to 3 February, 2016, 2017. Information on moult was derived from feathers found under the roosts. Feathers were removed from the roost at each visit.

**Feather measurements**

Barn swallows have tail lengths that are clearly bimodally distributed by sex and with HY birds having shorter tail length than adults (Møller 1994). We used the term “duration of feather moult” as the length of feathers divided by daily increments of feather growth rate (e. g. Grubb 2006). If daily growth increments reflect growth rate (Grubb 2006), then the sum of the length of all increments must be the length of the feather divided by the daily feather growth increment. Both male and female barn swallows and nestlings have white spots on their tail feathers (Møller 2020). Spot size is significantly larger in males than in females and in adults than in juveniles (Kose and Møller 1999). This criterion is sufficient for correct assignment. Again, our approach was to use tail feathers to quantify the proportion of adults vs HY birds in the sampled population. We measured the length and the width of feathers and the area of white spots on the tail feathers to the nearest 0.01 mm using a Mitutoyo digital calliper. Length of spots times width of spots was used as an estimate of spot area. These measurements of spot area allowed distinction between juveniles and adults (GLM with normally distributed data and an identity link function (LR = 7.80, df = 1. P = 0.005, mean (SE) for juveniles (5.68 (0.02))) and adults (5.97 (0.03)). We recorded the length of daily growth increments of wing and tail feathers (Grubb 2006) by holding the feather against a lamp over a piece of white paper placed on top of a styrofoam box. An insect pin was inserted at the start of a growth bar in the middle of the feathers and again at the end of the fifth growth bar because the width of five growth bars rather than say one single growth bar minimizes the magnitude of measurement errors. Anders Pape Møller subsequently measured the distance between the two holes at the start and the end of the five growth bars on the paper with a digital calliper with a precision of 0.01 mm. A total of 14 feathers were measured on two different days without knowledge of the first measurements to test for possible bias. There was a high repeatability (Falconer and Mackay 1996) with *F*_13, 14_ = 90.55, *r*^2^ = 0.99, P < 0.0001 and the repeatability *R* (SE) = 0.98 (0.02). Duration of moult in days was estimated as feather length divided by mean length of growth bars (Møller and Nielsen 2018). This conservative estimate revealed when these birds were moulting. The timing of moult was on average 64 days (1 = 1^st^ October), SE = 1.21, range 19 to 174, N = 561 feathers.

**Stable isotopes**

We restricted our isotopic assignment exercise to primary feathers for each of the two years for which samples were available from feathers moulted from the town of Aveiro (40º38' N, 8º39' W), Portugal. The seasonal phenology of occurrence of barn swallows at the roost was similar in 2015-2016 and 2016-2017 We prepared feather samples in the Environment Canada stable isotope laboratory at the National Hydrology Research Centre in Saskatoon, Canada. Each feather sample, representing the distal region of the vane of the feather, was soaked for 5 hours in 2:1 chloroform: methanol solution, then rinsed and dried in a fume hood for 48 h (Møller et al. 2018). The impact of exchangeable hydrogen on isotopic measurements was corrected using the comparative equilibration method described by Wassenaar and Hobson (2003). Within analytical runs, Environment Canada keratin reference standards CBS (Caribou hoof: -197‰) and KHS (Kudu horn: -54.1‰) were used to calibrate samples. Stable hydrogen isotopic measurements were performed on H_2_ gas derived from high-temperature (1350°C) flash pyrolysis of 350±10 μg distal vane feather subsamples in silver capsules. Resulting H gas was separated in a Eurovector 3000 (Milan, Italy) elemental analyser and introduced into an Isoprime (Crewe, UK) continuous flow isotope ratio mass spectrometer. All results are reported for non-exchangeable H expressed in the typical delta (δ) notation, in units of per mil (‰), and normalized on the Vienna Standard Mean Ocean Water – Standard Light Antarctic Precipitation (VSMOW-SLAP) scale (Wassenaar and Hobson 2006). Based on within-run replicate (N = 5) measurements of laboratory keratin standards, we estimated measurement precision to be ± 2‰.

Individual birds were assigned to moult localities where they annually change their feathers using a likelihood-based assignment method (Royle and Rubenstein 2017). Details of this approach using a probabilistic framework are described elsewhere (Hobson et al. 2012). Briefly, we employed algorithms relating variation in *δ*^2^H_f_ to variation in the amount-weighted mean *δ*^2^H of precipitation (*δ*^2^H_p_; Hobson et al. 2012; Bowen et al. 2005). Those algorithms were based on the use of the former keratin reference standard values CBS and KHS matching our laboratory reference values and so would give identical assignment if recently modified keratin standard values and associated calibrations (e.g. CBS: -157‰, KHS: -35.3‰) were used. Based on Hobson et al. (2012), we considered two possible feather-to-precipitation calibration algorithms in order to create an expected δ^2^H_f_ isoscape. We took this approach because such calibration relationships have not been explicitly derived for barn swallows and so we wanted to compare results from potentially competing algorithms based on North American and European avian isotopic datasets. The first was the passerine insectivore relationship *δ*^2^H_f_ = −17.6 + 0.95 δ^2^H_p_ based on North American feather datasets including the mean growing season average δ^2^H_p_ (Hobson et al. 2012). The second was provided by Prochazka et al. (2013) for Eurasian Reed Warbler (*Acrocephalus scirpaceus*) *δ*^2^H_f_ = −10.9 + 1.28 *δ*^2^H_p_ including mean annual precipitation. To limit assignment results to biologically plausible origins, we used a combination of digital range maps for the breeding grounds (BirdLife International & NatureServe 2004) and our knowledge of likely wintering regions in sub Saharan West Africa (De Bont 1957; Rudebeck 1957; Herroelen 1960; Broekhuysen and Brown 1960; Dowsett 1966; Mendelsohn 1973; Francis 1980; Møller et al. 1995; Loske and Lederer 1988). We clipped the calibrated isoscapes to the respective breeding and wintering ranges using functions in the “raster” package (Hijmans 2016) in the R statistical computing environment version 3.4.0 (Hijmans 2016). For each individual sample (bird), we assessed the likelihood that each cell in the calibrated isoscape represented a potential origin for that individual using the normal probability density function and applied a 2:1 odds ratio criterion for including each pixel of the map as a potential source (1) or not (0). Probability of origin surfaces were combined for all individuals in a population, whereby pixel data reflected how many individuals at a given pixel were included in a final population depiction.

**Figure S1.** Phenology of barn swallows at the winter roost in Aveiro, Portugal during the winters 2015-2016 and 2016-2017. The curves are the loess smoother curves to facilitate interpretation of data.

**Electronic Supplementary Table S1.** Bird ID and supplementary δ^2^H data (in ‰) for feathers of barn swallows *Hirundo rustica* moulting at Aveiro, Portugal, 2016-2017. Note that for consistency, only primary feathers were used in assignment to origins as depicted in Figure 1 of the main text.

| ID | δ^2^H | Feather |
| --- | --- | --- |
| 1 | -58.7 | Primary |
| 2 | -58.2 | Primary |
| 3 | -53.1 | Primary |
| 4 | -46.1 | Secondary |
| 5 | -57.7 | Secondary |
| 6 | -27.4 | Secondary |
| 7 | -71.1 | Primary |
| 8 | -57.6 | Primary |
| 9 | -55.2 | Primary |
| 10 | -54.5 | Primary |
| 11 | -47.4 | Primary |
| 12 | -77.3 | Primary |
| 13 | -50.2 | Primary |
| 14 | -50.6 | Tertiary |
| 15 | -50.6 | Primary |
| 16 | -44.9 | Primary |
| 17 | -51.4 | Primary |
| 18 | -47.3 | Primary |
| 19 | -58.3 | Primary |
| 20 | -47.1 | Primary |
| 21 | -91.8 | Primary |
| 22 | -78.7 | Primary |
| 23 | -79.9 | Primary |
| 24 | -52.8 | Primary |
| 25 | -50.9 | Primary |
| 26 | -50.5 | Primary |
| 27 | -48.3 | Primary |
| 28 | -48.3 | Primary |
| 29 | -68.8 | Primary |
| 30 | -47.1 | Primary |
| 31 | -47.6 | Primary |
| 32 | -46.4 | Primary |
| 33 | -55.0 | Primary |
| 34 | -49.2 | Tertiary |
| 35 | -42.5 | Tertiary |
| 36 | -58.4 | Primary |
| 37 | -43.7 | Primary |
| 38 | -58.7 | Tertiary |
| 39 | -56.9 | Primary |
| 40 | -45.6 | Primary |
| 41 | -40.8 | Primary |
| 42 | -42.2 | Primary |
| 43 | -40.8 | Primary |
| 44 | -61.0 | Tertiary |
| 45 | -58.6 | Tertiary |
| 46 | -59.3 | Primary |
| 47 | -58.9 | Primary |
| 48 | -65.7 | Primary |
| 49 | -61.1 | Primary |
| 50 | -49.4 | Primary |
| 51 | -49.1 | Primary |
| 52 | -74.2 | Primary |
| 53 | -61.7 | Primary |
| 54 | -67.9 | Primary |
| 55 | -62.7 | Primary |
| 56 | -69.5 | Primary |
| 57 | -62.2 | Primary |
| 58 | -51.7 | Primary |
| 59 | -47.8 | Primary |
| 60 | -71.4 | Primary |
| 61 | -66.2 | Primary |
| 62 | -71.4 | Primary |
| 63 | -54.9 | Primary |
| 64 | -66.9 | Primary |
| 65 | -64.6 | Primary |
| 66 | -68.6 | Primary |
| 67 | -64.3 | Primary |
| 68 | -70.9 | Primary |
| 69 | -63.8 | Primary |

**Literature Cited**

BirdLife International, NatureServe. 2004. BirdLife International, Cambridge, UK.

http://datazone.birdlife.org/species/factsheet/barn-swallow-hirundo-rustica/).

Bowen GJ, Wassenaar LI, Hobson KA. 2005. Global application of stable hydrogen and oxygen isotopes to wildlife forensics. *Oecologia* **143**:337-348.

Falconer DS Mackay TFC. 1996. *Introduction to Quantitative Genetics*. 4th edn. (Longman, New York, NY.

Francis DM. 1980. Moult of European swallows in Central Zambia. *Ringing Migration* **3**:4-8.

Grubb TC. Jr. 2006. *Ptilochronology.* Oxford University Press, New York, N

Hijmans RJ. 2016. *raster: Geographic Data Analysis and Modeling*. R package version 2.5-8 <https://CRAN.R-project.org/package=raster>.

Hobson KA., Van Wilgenburg SL, Wassenaar LIand K. Larson. 2012. Linking hydrogen (*δ*^2^H) isotopes in feathers and precipitation: Sources of variance and consequences for assignment to global isoscapes. *PLoS ONE* **7**(4): e35137. doi:10.1371/journal.pone.0035137

Loske K-H, Lederer W. 1988. Moult, weight and biometrical data for some Palaeartic passerine migrants in Zambia. *Ostrich* **59**:1-7.

Mendelsohn JH. 1973. Some observations on age rati2-19o, weight and moult in the European swallow, *Hirundo rustica* L. in the central Transvaal (Aves: Hirundinidae). *Ann. Transvaal Mus*. **6**:79-89.

Møller AP, Magnhagen C, Ulfstrand A, Ulfstrand S*.* 1995. Phenotypic quality and molt in the barn swallow, *Hirundo rustica*. *Behav Ecol* **5**:242-249.

Møller AP, Nielsen JT. 2018. The trade-off between rapid feather growth and impaired feather quality increases risk of predation. *J Ornithol*. **159**:165-171.

Prochazka P, Van Wilgenburg SL, Neto JM, Yosef R, Hobson KA. 2013. Using stable hydrogen isotopes (δ^2^H) and ring recoveries to trace natal origins in a Eurasian passerine with a migratory divide. *J. Avian Biol*. **44**:541-550.

Royle JA, Rubenstein D R. 2004. The role of species abundance in determining breeding origins of migratory birds with stable isotopes. *Ecol. Appl*. **14**:1780–1788.

van Nus T, Neto JM. 2017. Urban roost of wintering barn swallows *Hirundo rustica* in Aveiro, Portugal. *Ardea* **105**:73-78.

Wassenaar LI, Hobson KA. 2003. Comparative equilibration and online technique for determination of non-exchangeable hydrogen of keratins for use in animal migration studies. *Isotop. Environ Health* *Sci*. **39**:211–217.

Wassenaar LI, Hobson KA. 2006. Stable-hydrogen isotope heterogeneity in keratinous materials: Mass spectrometry and migratory wildlife tissue subsampling strategies. *Rapid Comm. Mass Spectrom*. **20**:16.
